# Genome-wide association study discovered favorable single nucleotide polymorphisms and candidate genes associated with ramet number in ramie (*Boehmeria nivea* L.)

**DOI:** 10.1186/s12870-018-1573-1

**Published:** 2018-12-12

**Authors:** Kunmei Chen, Mingbao Luan, Heping Xiong, Ping Chen, Jikang Chen, Gang Gao, Kunyong Huang, Aiguo Zhu, Chunming Yu

**Affiliations:** Institute of Bast Fiber Crops, Chinese Academy of Agricultural Sciences, No. 348, West Xianjiahu Road, Changsha, 410205 Hunan Province China

**Keywords:** Ramie, Ramet number, Genome-wide association study, Significant SNPs, qPCR

## Abstract

**Background:**

Ramie (*Boehmeria nivea* L.) is one of the most important natural fiber crops and an important forage grass in south China. Ramet number, which is a quantitative trait controlled by multigenes, is one of the most important agronomic traits in plants because the ramet number per plant is a key component of grain yield and biomass. However, the genetic variation and genetic architecture of ramie ramet number are rarely known.

**Results:**

A genome-wide association study was performed using a panel of 112 core germplasms and 108,888 single nucleotide polymorphisms (SNPs) detected using specific-locus amplified fragment sequencing technology. Trait-SNP association analysis detected 44 significant SNPs that were associated with ramet number at *P* < 0.01. The favorable SNP Marker20170–64 emerged at least twice in the three detected stages and was validated to be associated with the ramie ramet number using genomic DNA polymerase chain reaction with an F_1_ hybrid progeny population. Comparative genome analysis predicted nine candidate genes for ramet number based on Marker20170–64. Real-time quantitative polymerase chain reaction analysis indicated that six of the genes were specific to upregulation in the ramie variety with high ramet number. These results suggest that these genes could be considered as ramet number-associated candidates in ramie.

**Conclusions:**

The identified loci or genes may be promising targets for genetic engineering and selection for modulating the ramet number in ramie. Our work improves understanding of the genetics of ramet number in ramie core germplasms and provides tools for marker-assisted selection for improvement of agricultural traits.

**Electronic supplementary material:**

The online version of this article (10.1186/s12870-018-1573-1) contains supplementary material, which is available to authorized users.

## Background

Ramie (*Boehmeria nivea* L.), which is native to China and commonly known as China grass, is one of the oldest fiber crops worldwide [1]. It has a history of over 4000 years as a fiber crop in China and has been popularly used as animal feed and for the phytoremediation of heavy metal-contaminated farmlands over the past decades. The superior fiber obtained from its woody stem is long and highly durable, pure white in color, and silky in texture, with a high degree of hygroscopicity and superior heat dissipation. These superior characteristics make ramie a highly versatile and useful natural raw textile material. Generally, raw ramie fiber can be preserved for 20–30 years. The peak period of global ramie production occurred between 2001 and 2007, and this provided many raw ramie stocks, that have met the processing and consumption demand in recent years. The total production of ramie worldwide has declined in recent years, whereas the consumption has increased annually (http://www.fao.org/faostat/en/#home), indicating an increasing requirement for raw ramie fiber. High-end textile products fabricated with natural fiber have become increasingly popular and, consequently, the demand for ramie raw textile material could be predicted to rise in the future. Ramie has a high tolerance and strong ability to absorb multiple heavy metals such as cadmium [2], lead [3], and arsenic [4] from contaminated soil. It is therefore considered to be one of the most potentially useful plants for remediation of heavy metal contaminated land. Plants with high biomass have an advantage in heavy metal absorption and, thus, improving the biomass of ramie is meaningful. In addition, because ramie is used as animal feed, high biomass is beneficial for promoting the development of the ramie feed industry. Taken together, these factors indicate the considerable significance of improving the fiber and biomass yield of ramie.

The number of ramet (in clonal plants), or tiller (in monocotyledons), is an important factor influencing crop yield [5]. For instance, the tillering ability is one of the most important traits in rice since it can have a significant effect on the future production of panicles [6], which in turn is highly correlated with grain yield [7]. For perennial plants, the ramet formation ability is an important indicator of their potential biomass production. In *Eupatorium adenophorum*, a perennial clonal plant, the biomass increases with increasing ramet number [8]. Ramie is a perennial herbaceous plant, and its ramet number is one of the most important targets in ramie breeding. Compared with the plant height and leaf number, the ramet number is the main factor affecting ramie biomass [9]. Bai et al. [10] showed that there is a significant correlation between fiber yield and ramet number in ramie. Therefore, improving the ramet number in ramie is important for the biomass or the fiber product.

Single nucleotide polymorphism (SNP) refers to the DNA sequence polymorphism caused by the variation of single nucleotide at the genomic level. SNP often occurs in the genome and is the most abundant and stable form of genetic variation, which provide valuable markers for the study of agronomic traits in crops [11]. Specific locus amplified fragment sequencing (SLAF-seq) is a relatively new high-resolution strategy that can fast, accurately, efficiently, and economically develop large-scale SNP and InDel markers. The flowchart of SLAF-seq technology comprises: i) pre-design scheme for SLAF selection, ii) SLAF-seq library construction, and iii) high-throughput sequencing and genotyping [12].

Ramet or tiller number per plant is a quantitative trait controlled by multigenes, and is reported to be equally regulated by additive and dominant gene effects [13]. Genome-wide association study (GWAS) is a powerful tool for complex trait dissection in plants [14]. Compared to biparental linkage mapping, GWAS has the advantages of high resolution, cost-efficiency, and not requiring the creation of a mapping population. With the rapid development of DNA and RNA-sequencing technologies, high-density genotyping with single nucleotide polymorphisms (SNPs) has become easily accessible, enabling GWAS to be performed in many plants including maize [15], rice [16], cotton [17], canola [18], sorghum [19], foxtail millet [20], and *Arabidopsis* [21].

Significant SNPs and candidate genes associated with quantitative traits have been detected in a large variety of plants using GWAS. In cotton, one favorable SNP associated with verticillium wilt resistance was identified from 17 significant SNPs detected using GWAS based on SLAF-seq technology, and 22 candidate genes for verticillium wilt resistance were predicted based on a favorable SNP [22]. Six candidate genes with pleiotropic effects on stalk cell wall components in maize have been identified using the GWAS method [23]. Furthermore, in *Arabidopsis*, a gene associated with leaf arsenic accumulation was identified using GWAS [24]. Previously, five QTLs were identified in an F_2_ agamous line population of ramie using 114 SSR markers, although they were not confirmed in a different population [25]. To better understand the genetics of ramie ramet formation, further investigations are needed.

To investigate the genetic components underlying the natural variation in ramie germplasms and discover favorable SNPs associated with ramet number, we performed a GWAS using SLAF-seq technology on 112 ramie core germplasms. The favorable SNPs associated with ramet number were further validated in a hybrid progeny population using a genome DNA polymerase chain reaction (PCR) strategy. Furthermore, the candidate genes associated with ramet number are discussed. This study will be helpful in clarifying the genetic structure of the ramie core germplasms and providing information about candidate quantitative trait loci (QTL) and genes that control the ramet number in ramie.

## Material and methods

### Ethics statement

The collection of ramie specimens used in this study were planted in our scientific research field, which is owned by our institution. Therefore, no specific permissions were required for using these specimens.

### Plant materials and statistics of ramet number

A total of 112 core germplasms of ramie planted in the Institute of Bast Fiber Crops, Chinese Academy of Agricultural Sciences were used for SNP development in this study. These germplasms were collected from China, India, Indonesia, Brazil, and Cuba (Additional file 1). An F_1_ hybrid progeny population and its parents (two ramie varieties: Zhongzhu NO.1 and Hejiangqingma with high and low ramet numbers, respectively), were used for SNP validation using PCR. All ramie plants were propagated by asexual propagation, and planted in a field (N 28° 21′, E 112° 59′) at Changsha, China, in 2014. For each core germplasm, four plants were taken from the side branches of the same parent plant, and these were planted in a row with four plots (one plant per plot), with a distance of 40 cm between plots and 60 cm between rows. Each F_1_ hybrid progeny was planted with one plant per plot, four plots per row, with 40 cm between plots and 60 cm between rows. For the 112 core germplasms and the F_1_ hybrid progeny, three biological replicates were set up with a randomized block design for each replicate.

The ramet number of the core germplasm was recorded in May, August, and November 2016, when the plants had grown to 80 cm in height. The ramet number of the F_1_ hybrid progeny population was recorded in March and November 2017.

### SNP genotyping and quality control

Fresh leaves of four plants were mixed as one sample for each core germplasm. Total genomic DNA was extracted from each sample using the modified cetyltrimethylammonium bromide method described by Luan et al. [26]. DNA quality and quantity were determined using a Nanodrop 2000 spectrophotometer (Thermo Fisher Scientific, Wilmington, DE, USA) and an Agilent 2100 Bioanalyzer (Agilent Technologies, Waldbronn, Germany). Quantified DNA was diluted to 100 ng μL^− 1^ for SLAF sequencing. The SLAF library was constructed as previously described [12] with slight modifications. The genome of *Cannabis sativa* (ftp://ftp.ncbi.nlm.nih.gov/genomes/all/GCA/003/417/725/GCA_003417725.2_ASM341772v2/) was used as a reference to electronically predict the result of enzyme digestion and determine an optimal restriction enzyme solution according to the following criteria: (1) the proportion of restriction fragments in the repeated sequence is as low as possible, (2) restriction fragments are distributed as evenly as possible in the genome, (3) simulated fragments align uniquely to the reference genome, and (4) a high number of SLAF tags [27]. Furthermore, the restriction enzyme combination of RsaI and HaeIII was selected. Plant DNA was digested with a combination of RsaI + HaeIII (NEB, Ipswich, MA, USA) to obtain the SLAF tags (defined as enzyme fragment sequences of 264–394 bp), followed by dual-index paired-end adapter ligation, PCR amplification, and target fragment selection for the SLAF library construction. The selected fragments and a control (*Oryza sativa ssp. japonica*) were sequenced using the Illumina Hi-Seq 2500 sequencing platform (Illumina Inc., San Diego, CA, USA) at Biomarker Technologies Corporation in Beijing (http://Biomarker.com.cn/). The raw data were assessed using the dual-index to obtain reads for each sample. After filtering out adapter reads, the sequence quality was evaluated by analyzing the guanine-cytosine content and the Q30 quality score (Q = − 10 × log^e^_10_, indicating a 0.1% chance of an error and, thus, 99.9% confidence) quality score. Effectiveness and accuracy were evaluated using enzyme-cut rate information from the control. All reads were checked using cluster analysis based on sequence similarity. Reads from different samples were classed into one set named the SLAF tag. A SLAF tag that exhibited differences in sequences from other samples was defined as polymorphic. Using the Burrows-Wheeler Alignment tool software [28], the sequenced reads were compared with the reference tag, which was the deepest sequence in each SLAF tag. The GATK38 [29] and SAMtools [30] packages were used to perform SNP calling, and the SNPs obtained by both of these methods were treated as reliable SNPs. Finally, the reliable SNPs were filtered out with integrity > 0.8 and MAF > 0.05.

### Population structure and kinship analysis

The population structure of the 112 core germplasms was analyzed using an admixture software [31]. The number of simulation subgroups (*K* value) was set from 1 to 10. The statistic *ΔK* was calculated using STRUCTURE HARVESTER [32] (http://taylor0.biology.ucla.edu/structureHarvester/). The *ΔK* was set as the determinant factor for evaluating the optimal value of *K* [33]. The Q-matrix was obtained using the CLUMPP software [34]. The phylogenetic tree of the 112 core germplasms was constructed using MEGA 5.1 with the neighbor-joining (NJ) method (1000 bootstraps) [35]. The kinship (K) matrix was estimated using SPAGeDi version 1.4b [36].

### GWAS

Phenotype–genotype association analysis and allele effect calculations were performed using the TASSEL software. Two models, the general linear model (GLM) adjusted using the O-matrix (GLM [Q]) and the mixed linear model (MLM) correcting for both Q-matrix and K-matrix (MLM [Q + K]), were used to reduce errors from population structure and relative kinship. Those with *P* < 0.01 adjusted by the Bonferroni method were defined as significant trait-associated SNPs.

### Validation of favorable SNPs

The F_1_ hybrid progeny population described above, consisting of 241 lines, was used for validation of the favorable SNPs via a genomic DNA PCR strategy with special primers (Additional file 2) designed based on SNP sequence according to the method described by Chen et al. [37]. DNA samples were extracted from young leaves of each line using a DNeasy plant mini kit (Tiangen, Beijing, China). PCR amplification was performed in a 20 mL reaction volume consisting of 1 × EasyTaq buffer, 0.2 mmol/L dNTPs, 0.5 mmol/L of each of the forward and reverse primers, 2.5 units of EasyTaq DNA Polymerase (Transgen Biotech, China), 100 ng of DNA template, and an appropriate amount of sterile double-distilled water. The amplification schedule was run as follows: an initial denaturation at 95° for 5 min, followed by 33 cycles of 95° for 30 s, 58° for 45 s, and 72° for 1 min. PCR products were separated using 8% polyacrylamide gels, and silver staining was conducted according to the method of Luan et al. [26]. Molecular weights were estimated using a DNA marker (DNA Marker 2000, BioTeke Co., Beijing, China). Clear amplified bands were recorded as 1 and the absence of bands was recorded as 0. The products of clean bands were sequenced at Biomarker Technologies Corporation to ensure the authenticity of the target sequences.

Spearman correlation analysis using IBM SPSS Statistics 19.0 (IBM, NY, USA) was performed to determine if the ramet number in the F_1_ hybrid progeny had any correlation with the SNPs. A difference between means was considered statistically significant at *P* < 0.05.

### Identification of candidate genes and quantitative PCR (qPCR) analysis

Sequences of favorable SNPs were used to blast the ramie genome [38], and genes located in the region 150 kb upstream or downstream of the ramet number associated SNPs were identified as candidate associated genes.

Three replicates of each of two germplasm plants (Chuanzhu NO.2 and Quxianzhuma, Additional file 1) were used for the qPCR analysis. For the expression of candidate genes, total RNA was extracted from the leaf and stem bark of each germplasm using a plant RNA purification kit (Tiangen, Beijing, China). Briefly, 0.5 μg total RNA was used to synthesize cDNA (in 10-mL reaction volumes) using a PrimeScript RT perfect real-time reagent kit (TaKaRa, Japan). Subsequently, the cDNA was diluted four times. The qPCR analysis was performed using a Lightcycler 480 engine (Roche, Germany) using the 2 × T5 Fast qPCR Mix (SYBR Green I, TSINGKE Biological Technology, Beijing, China) in a 20-mL reaction volume consisting of 10 μL 2 × T5 Fast qPCR mix, 0.4 μmol/L of each of the forward and reverse primers (listed in Additional file 3), 2 μL diluted cDNA, and an appropriate amount of sterile double-distilled water. The PCR conditions consisted of an initial denaturation step at 95° for 1 min, followed by 40 cycles of denaturation at 95° for 10 s, annealing at 60° for 5 s, and extension at 72° for 15 s. The relative expression was calculated using the 2^-ΔΔCt^ method [39] using the housekeeping gene *18S* as an internal control.

## Results

### Phenotypic characteristics of ramet number

For the 112 core germplasms, the ramet number of each germplasm varied widely in each season (Table 1), ranging from 2.00 to 13.83, with an average of 7.66, in May 2016; from 5.00 to 18.00, with an average of 9.48, in August 2016; and from 4.33 to 21.50, with an average of 11.68, in November 2016. These data illustrated the large variation amplitude of the core germplasm population, indicating that it could be an excellent population for marker-trait GWAS.

**Table 1:** Phenotypic variation of ramet number in association analysis and validation population

Population	Detected stage	Mean	SD	Min	Max	CV (%)
Association analysis population	May, 2016	7.66	2.34	2.00	13.83	30.49
	August, 2016	9.48	2.61	5.00	18.00	27.56
	November, 2016	11.68	3.15	4.33	21.50	26.97
Validation population	March, 2017	5.29	1.96	1.00	10.00	37.14
	November, 2017	8.29	3.23	2.00	18.00	38.91

*SD* standard deviation, *CV* coefficient of variation

For the hybrid progeny population (validation population), the ramet number of 241 lines was analyzed in two seasons (in March and November 2017). The variation in ramet number was 10-fold in March 2017, from 1 to 10, and 9-fold in November 2017, from 2 to 18 (Table 1). The variation of the ramet number in the two seasons showed a normal distribution curve (Additional file 4). The large variation in ramet number in the validation population was an advantage for validation of favorable SNPs.

### SNP-based genotyping of ramie accessions

To finely map the ramet number associated genes and investigate beneficial haplotypes in the ramie germplasm, a haplotype map of the 112 core germplasms was constructed using the SLAF-seq approach. In total, over 364.29 Mb reads were generated for the 112 genotypes (Table 2). Approximately 2,458,923 high-quality SLAF tags were identified from the total reads, and 336,623 of the high-quality SLAF tags showed high polymorphism. The SLAF tags used to call the SNPs had an average depth of 10.89-fold per individual among the 112 germplasms. In total, 1,113,711 SNPs were initially called for this set of lines; after further exclusion at MAF > 0.05 and integrity > 0.8, 108,888 high-consistency SNPs were retained for the analysis. These SNPs were used to assess population structure and GWAS analysis.

**Table 2:** Summary of statistic data generated by specific-locus amplified fragment sequencing (SLAF-seq) technology

Name	Number
Total reads	364.29 Mb
High-quality SLAF tags	2,458,923
High polymorphism reads	336,623
High-consistency SNPs	108,888

*SNP* single nucleotide polymorphisms

### Population structure and kinship

**Fig. 1:**
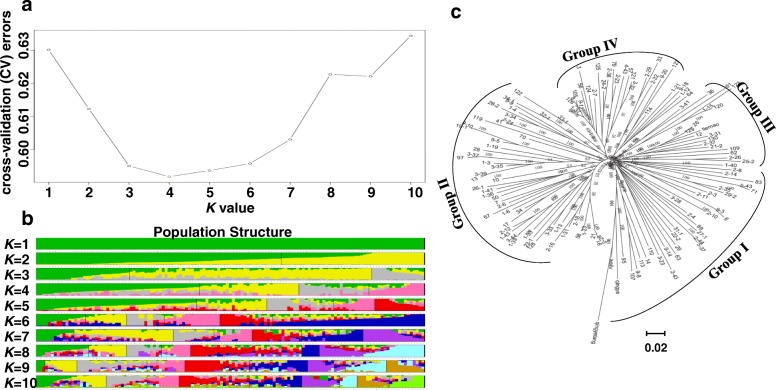
Population structure of 112 core germplasms of ramie. **a** *ΔK* values plotted from 1 to 10. **b** Population structure of the 112 core germplasms based on STRUCTURE, where *K* = 1 to 10. **c** Neighbor-joining (NJ) tree of 122 core germplasms based on Nei’s genetic distances

Considering the fact that the authenticity of QTL mapping could be affected by population structure, it is critical to understand the structure matrix in GWAS populations. In this study, the number of subgroups in the 112 core germplasms was estimated with two methods based on the genotypic database. First, the Bayesian clustering from *K* = 1 to 10 was calculated with the STRUCTURE software. The delta *K* value reached the lowest point at *K* = 4 (Fig. 1a), suggesting that the population could be divided into four subgroups (Fig. 1b, Additional file 1): Groups I, II, III, and IV. Secondly, an NJ phylogeny based on genetic distances also showed that the 112 core germplasms were outlined with four main clusters (Fig. 1c), which was consistent with the result of the STRUCTURE analysis, despite some accessions overlapping in the four clusters.

### Marker-trait GWAS

The GWAS was performed using GLM and MLM models with 108,888 high-consistency SNPs. To confirm the potentially significant SNPs associated with ramet number, genotypes from three stages of the 112 core germplasms were analyzed. For the GLM model, a total of 2553, 2256, and 4288 significant SNPs were detected at *P* ≤ 0.01 in May, August, and November in 2016, respectively, and 44 common significant SNPs were shown in all three stages (Additional files 5 and 6). For the MLM model, 1067, 815, and 4049 significant SNPs were found at *P* ≤ 0.01 in the three stages, and four of the SNPs (Marker38532–124, Marker54845–111, Marker57363–43, and Marker75993–18) emerged in all three stages at the same time. Some of the detected significant SNPs emerged at least twice in the same stages; for example, the SNP (Marker20170–64) generated using the MLM model emerged twice in May.

### Identification and validation of favorable SNPs associated with ramet number

**Fig. 2:**
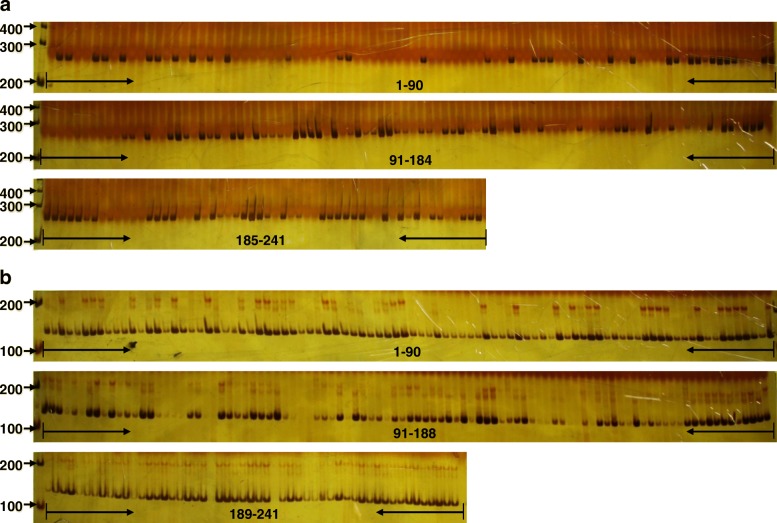
Verification of favorable single nucleotide polymorphisms (SNPs) in an F_1_ hybrid progeny population of ramie using genomic DNA polymerase chain reaction (PCR). **a** Results of PCR amplified using specific primers of Marker20170–64. The target product size was 241 bp. The first lane on the left is DNA marker. The numbers 200, 300, and 400 denote 200, 300, and 400 bp, respectively. **b** PCR amplification using specific primers of Marker142939–43. The target product size was 125 bp. The numbers 1–241 denote 241 lines of the F_1_ hybrid progeny population. The first lane on the left is DNA marker. The numbers 100, 200, and 300 denote 100, 200, and 300 bp, respectively

To understand the effects of allelic variation on the ramet number, 20 significant SNPs, which emerged at least twice in the three stages analyzed, were identified as favorable alleles. An F_1_ hybrid progeny population of 241 lines and its parents were used to verify the authenticity of the favorable SNPs using genomic DNA PCR with specific primers (Additional file 2). First, DNA of the parents was used as a template for amplification with the 20 pairs of primers, and the results showed that two (Marker20170–64 and Marker142939–43) of the favorable SNPs amplified a product in one but not in the other parent (Additional file 7). Five of the favorable SNPs (Marker70439–41, Marker13742–63, Marker15847–131, Marker21174–109, and Marker18389–39) amplified a product in both parents. The remaining 13 favorable SNPs did not amplify any product in either parent. After verifying the amplification products by sequencing, Marker20170–64 and Marker142939–43 SNPs were selected for further amplification using the DNA samples of the 241 lines as templates. As shown in Fig. 2, amplification was successful in 126 of the 241 lines using Marker20170–64, which was close to the ratio of 1:1 (Table 3), whereas 222 of the 241 lines were amplified using Marker142939–43. The results of the correlation analysis between the trait (ramet numbers of the 241 lines) and the amplification results, suggests that the SNP Marker20170–64 was associated with the ramet number in ramie (Table 4).

**Table 3:** Test for goodness-of-fit (χ^2^) between ramet number and PCR results in validation population

Amplification band type	Ramet number (*O*)	Ramet number (*E*)	*O*-*E*	(|*O*-*E*|-1/2)^2^/*E*
None	115	120.5	−5.5	0.2075
Exist	126	120.5	5.5	0.2075
Total	241	241	0	0.4150

*O* denotes observed value, *E* denotes theoretical value

**Table 4:** The correlation (*r*) between single nucleotide polymorphisms (SNPs) and ramet number

Trait	Marker20170	Marker142939
Ramet number in March 2017	− 0.201^a^	0.039
Ramet number in November 2017	−0.094	0.013

^a^denotes significant difference between phenotypes and genotypes at *P* < 0.01

### Candidate gene identification and expression analysis

As validated above, the SNP Marker20170–64 may be a major genetic locus responsible for ramet number in ramie. Thus, the haplotype block structure was investigated within 150 kb on either side of Marker20170–64 to determine candidate genes. A total of nine genes were found in the region of Marker20170–64. Bioinformatic analysis showed that three of these genes lacked any definite annotation for their biological functions and four genes were linked to biological pathways involved in plant growth and development (Additional file 3).

**Fig. 3:**
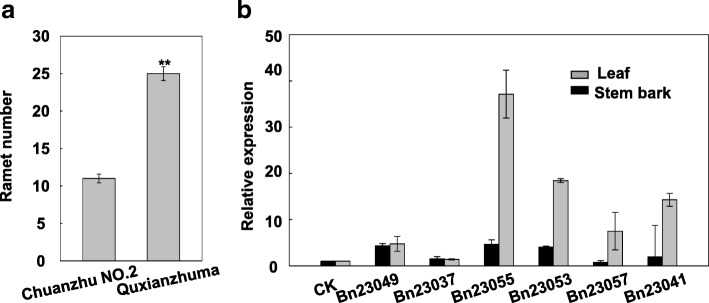
**a** Ramet number of two ramie varieties (Chuanzhu NO.2 and Quxianzhuma). ^**^ denotes statistically significant differences from the control at *P* < 0.01. **b** Expression of candidate genes (*Bn23049*, *Bn23037*, *Bn23055*, *Bn23053*, *Bn23057*, and *Bn23041*) in leaf and stem bark of Chuanzhu NO.2 and Quxianzhuma. The 2^-ΔΔCt^ method was used to calculate the relative expression level of target genes. The expression in Chuanzhu NO.2 was used as control (CK) and its value was set as 1. Data are means ± SE of three replicates. Each replicate consisted of material from three plants that grew to 40–50 cm in height

To determine the genes responsible for ramet number in ramie, a qPCR analysis was performed using two ramie varieties with significantly different ramet numbers (Fig. 3a). The results showed that six genes (*Bn23049*, *Bn23037*, *Bn23055*, *Bn23053*, *Bn23057*, and *Bn23041*) were upregulated in different tissues (leaf and stem bark) of the high ramet number genotype Quxianzhuma (Fig. 3b). Two (*Bn23037* and *Bn23053*) of these six genes showed functions in regulating root development, one (*Bn23049*) in regulating seed germination, and one (*Bn23041*) was associated with cell fate determination and maintenance of floral/inflorescence/shoot apical meristem identity.

## Discussion

### Application of GWAS in ramie

Ramet number is a significant quantitative trait in ramie, which is controlled by multigenes. Understanding the mechanism of ramet number determination through gene/SNP/QTL analysis and breeding high ramet number cultivars using marker-assisted selection are thought to be the most practical and effective strategies to manage this trait. However, it is difficult to reliably identify effective SNPs/QTLs associated with ramet number in ramie for various reasons, including the low genome coverage of the available molecular markers, the slight effects of most loci, the limited allelic segregation, and recombination of biparental populations in linkage mapping. In this study, we used an association population consisting of 112 core germplasms collected from China, Japan, India, Indonesia, and Cuba, for ramet number-associated SNP detection, which offered more historical recombination events, to overcome the limitations of biparental populations.

In this study, GWAS, which has been used to map complex quantitative traits in plants [21, 40–42], was performed for the first time in ramie to detect association between SNPs and quantitative traits. The power of GWAS depends on four main factors: the richness of genetic diversity, the veracity of trait acquisition, the marker density, and the statistical methods. The core germplasms used for the GWAS in our study were collected from different regions of China, representing the typical characteristics of various ramie varieties. Furthermore, they have high levels of genotypic and phenotypic diversity (Table 1), which is suitable for GWAS. Because ramet production in ramie can be affected by nutrient, harvest time, and other environmental factors [43, 44], phenotypic data from three stages were analyzed to ensure that the detected SNPs were reliable. Two models, GLM and MLM, were employed to reduce errors associated with population structure and relative kinship. Each step was verified to ensure that the significant SNPs identified in this study were reliable and reproducible.

### Verification of significant SNPs associated with ramet number

Some false positives may occur in plant GWASs because of genetic heterogeneity among different varieties, resulting in some SNPs being significant in one population but not in others [45]. Therefore, it is necessary to verify the significant SNPs detected using GWAS with different populations. In this study, 20 significant SNPs, which emerged at least twice in the three stages analyzed were identified as favorable alleles and verified using an F_1_ hybrid progeny population of 241 lines. The SNP (Marker20170–64) was found to be associated with ramet number in the hybrid progeny population, confirming its authenticity and demonstrating the need to verify significant SNPs detected by GWAS. Additionally, three loci (Marker20170–57, Marker20170–1, Marker 20,170–103) close to the Marker20170–64 were identified using SLAF-seq technology. All of these loci emerged at least twice in the three stages analyzed (Additional file 8), further confirming the authenticity of the Marker20170–64.

### Identification of candidate genes

In perennial plants such as ramie, *Potentilla anserina*, *Rubus saxatilis*, *Linnaea borealis*, and tussock, ramet number is a complex trait [46–48]. For instance, the size of the ramet population in Carex humilis increases with age; flowering ramets do not produce any offspring ramets, and larger parent ramets produce more and larger offspring ramets [47]. At the molecular level, many genes that control tiller number per plant have been cloned from diverse plant species. *PvSPL2*, an SBP-box transcription factor that affects lignin biosynthesis in switchgrass, predominantly modulates tiller initiation and stem elongation [49]. In rice, *OsHTD2*, which is involved in the strigolactone biosynthetic pathway, negatively regulated tiller bud outgrowth via the strigolactone pathway [50]. *OsIAA6*, a member of the rice *Aux/IAA* gene family, is involved in drought tolerance and tiller outgrowth [51]. These research findings show that there are multiple factors and pathways controlling ramet/tiller number in different plants. In this study, the PCR results in the validation population showed that the ratio of presence and absence of bands in the offspring with high and low ramet number was close to 1:1. These results indicate that ramie ramet number may be controlled by a single gene pair. However, the average ramet number of the validation population was normally distributed, which denotes that the ratio of low ramet number to high ramet number was not 1:1, indicating that ramie ramet number is a quantitative trait controlled by multiple genes. By comparing the Marker20170–64 to the ramie genome, we identified six genes that appeared to be related to ramet number, suggesting that ramet number is controlled by multiple genes in ramie.

The potential role of these six target genes in controlling ramet number was indicated by their up-regulated expression in the high ramet number genotype Quxianzhuma and their roles in the regulation of root development (*Bn23037* and *Bn23053*), seed germination (*Bn23049*), and cell fate determination and maintenance of floral/inflorescence/shoot apical meristem identity (*Bn23041*). A homologous gene of *Bn23037* in *Arabidopsis*, *AtERF4* (ERF, ethylene response factor), has a vital function in regulating leaf growth and development by responding to nutrition stress [52]. *AtTZF5*, whose CDS shows the highest homology with *Bn23049*, affects seed germination by controlling genes critical for abscisic acid and gibberellic acid response [53]. *AtRING1a*, a homologous gene of *Bn23041*, plays a primary role in the maintenance of meristem function by inhibiting the expression of a Class I KNOTTED-like homeobox transcription factor [54]. Plant hormones such as ethylene, abscisic acid, and gibberellic acid play a key role in controlling multiple aspects of plant growth and development, including tissue differentiation, root elongation, shoot branching, and flowering time [55]. The up-regulated expression of the six candidate genes may affect the changes of plant hormones and further regulate ramet development in ramie. However, because of the dearth of related literature on the ramet number of ramie, the biological function of the candidate genes identified in this study should be further verified using biological experiments. Taken together, the genes identified in our study could be used as candidate resources for the molecular improvement of ramet number in ramie.

## Conclusions

Using the GWAS method for the first time in ramie, we genotyped 112 ramie core germplasms and identified 44 SNPs significantly associated with ramet number across three stages and nine genes. Collectively, the identified SNPs and genes could be used as candidate resources for the molecular improvement of ramet number in ramie. In conclusion, our study provides technological strategies for quantitative trait studies of ramie.

## Additional files

Additional file 1: One hundred twelve core germplasms used for SNP genotyping. (XLSX 14 kb)

Additional file 2: qPCR primer sequences of favorable SNPs and reference gene. (XLSX 11 kb)

Additional file 3: Primers and functional description of candidate genes. (XLSX 11 kb)

Additional file 4: Normal distribution curve of the ramet number in hybrid progeny population in (a) dMarch 2017 and (b) November 2017. (PDF 20 kb)

Additional file 5: SNPs generated from GLM and MLM analysis when *P* < 0.01. (XLSX 364 kb)

Additional file 6: Common significant SNPs in GLM analysis and MLM analysis. (XLSX 11 kb)

Additional file 7: Results of polymerase chain reaction (PCR) amplification using specific primers of significant single nucleotide polymorphisms (SNPs) in parents of the F_1_ hybrid progeny population. M denotes 100 bp DNA Ladder, the bands from bottom to the top represent 100, 200, 300, 400, 500, 600, 700, 800, 900, 1000, and 1500 bp, respectively. The numbers 1–20 represent Marker70439–41, Marker13742–63, Marker29152–60, Marker42663–35, Marker59771–76, Marker142939–43, Marker15847–131, Marker121162–120, Marker20170–64, Marker21174–109, Marker130175–63, Marker18389–39, Marker12702–125, Marker178103–63, Marker21112–51, Marker24814–129, Marker31623–105, Marker39027–122, Marker73630–112, and Marker38532–124, respectively. For each SNP marker, amplifications from left to right represent three female (Hejiangqingma) and two male (Zhongzhu NO.1) parents. (PDF 45 kb)

Additional file 8: Additional SNP loci near the Marker20170–64. (XLSX 10 kb)

## Abbreviations

GLM: General linear model
GWAS: Genome-wide association study
MLM: Mixed linear model
NJ: Neighbor-joining
PCR: Polymerase chain reaction
qPCR: quantitative PCR
QTL: Quantitative trait loci
SLAF-seq: Specific-locus amplified fragment sequencing
SNPs: Single nucleotide polymorphisms
