# Supplementary figures and images for: Genome-wide association study discovered favorable single nucleotide polymorphisms and candidate genes associated with ramet number in ramie (Boehmeria nivea L.)
Source: BMC Plant Biol. 2018 Dec 12;18:345. doi: 10.1186/s12870-018-1573-1 (PMC6292125; doi:10.1186/s12870-018-1573-1)

## Additional file 4

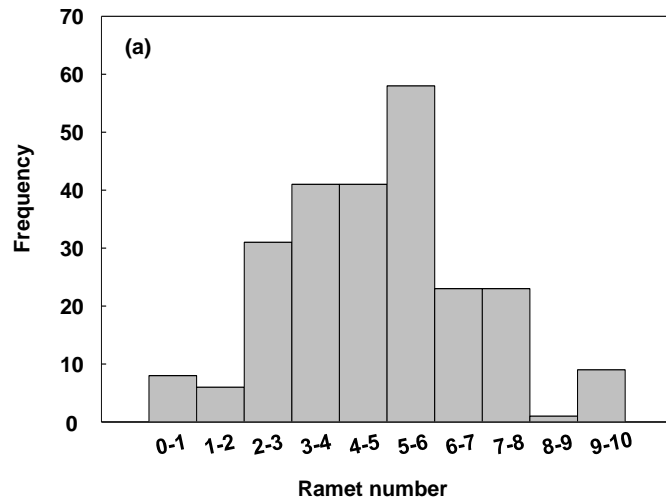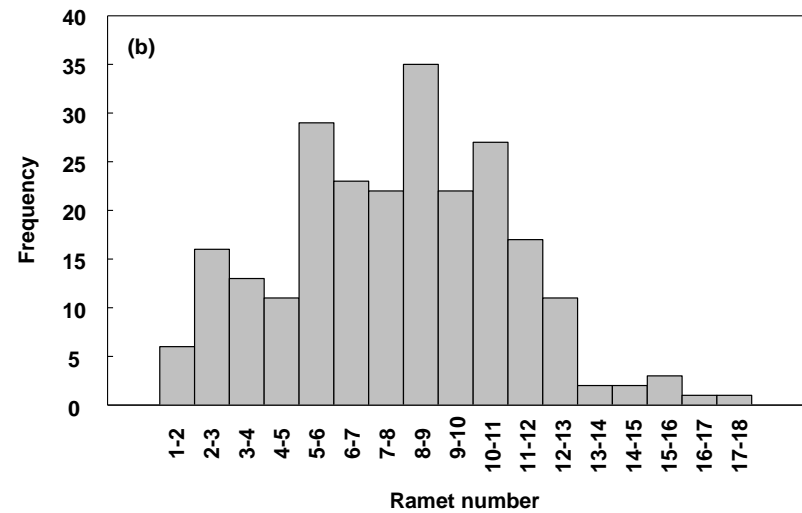

Supplement: Supplementary file 4 — Normal distribution curve of the ramet number in hybrid progeny population in (a) dMarch 2017 and (b) November 2017. (PDF 20 kb) [file 12870_2018_1573_MOESM4_ESM.pdf]

## Additional file 7

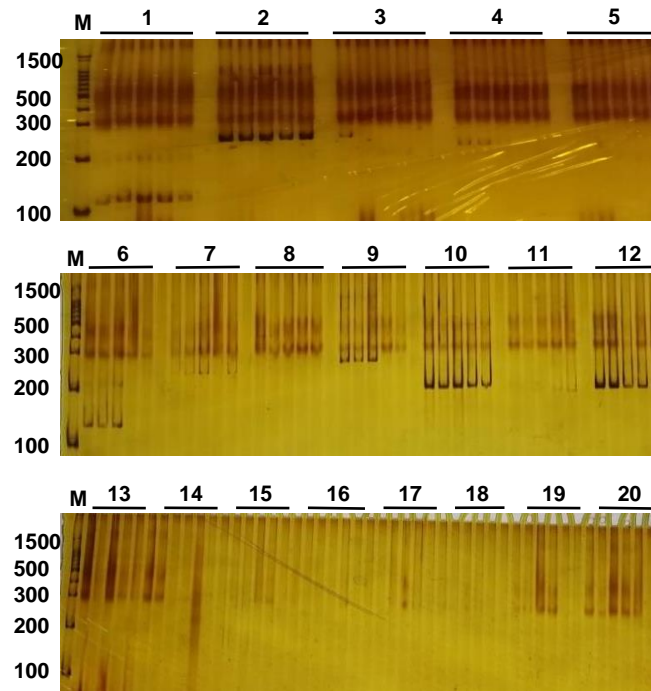

Supplement: Supplementary file 7 — Results of polymerase chain reaction (PCR) amplification using specific primers of significant single nucleotide polymorphisms (SNPs) in parents of the F1 hybrid progeny population. M denotes 100 bp DNA Ladder, the bands from bottom to the top represent 100, 200, 300, 400, 500, 600, 700, 800, 900, 1000, and 1500 bp, respectively. The numbers 1–20 represent Marker70439–41, Marker13742–63, Marker29152–60, Marker42663–35, Marker59771–76, Marker142939–43, Marker15847–131, Marker121162–120, Marker20170–64, Marker21174–109, Marker130175–63, Marker18389–39, Marker12702–125, Marker178103–63, Marker21112–51, Marker24814–129, Marker31623–105, Marker39027–122, Marker73630–112, and Marker38532–124, respectively. For each SNP marker, amplifications from left to right represent three female (Hejiangqingma) and two male (Zhongzhu NO.1) parents. (PDF 45 kb) [file 12870_2018_1573_MOESM7_ESM.pdf]
